# Molecular evolution of mosaic chromosome 18 copy-number alterations from gametes to hepatoblastoma

**DOI:** 10.1016/j.jhepr.2026.101862

**Published:** 2026-04-20

**Authors:** Elise Cendres, Marianna Cornet, Zoé Gautier, Aurore Pire, Noémie Urvoy, Guillaume Morcrette, Fatoumata Simaga, Anne Guimier, Christophe Chardot, Carmen Capito, Gudrun Schleiermacher, Julien Masliah-Planchon, Gaelle Pierron, Ilaria Taddei, Dominique Stoppa-Lyonnet, Jessica Zucman-Rossi, Serge Romana, Isabelle Aerts, Theo Z. Hirsch

**Affiliations:** 1 Université Paris Saclay, 91190, Gif-sur-Yvette, France; 2 Centre de Recherche des Cordeliers, Université Paris Cité, Sorbonne Université, INSERM, 75006, Paris, France; 3 Hôpital Necker-Enfants Malades, Université Paris Cité, Paris, France; 4 Fetopathology Department, Robert Debré Hospital, APHP, Paris Cité University, Paris, France; 5 Genetics Department, Institut Curie, Paris, France; 6 Service de Médecine Génomique des Maladies Rares, Hôpital Necker-Enfants Malades, AP-HP and Laboratory “Genetics of Developmental Disorders”, INSERM U1163, Université Paris Cité, Institut Imagine, Paris, France; 7 SIREDO Integrated Pediatric Oncology Center and U1330 INSERM, PSL Research University, Institut Curie, Paris, France; 8 PSL Research University, Paris, France; 9 Service de Médecine Génomique des Maladies Rares, AP-HP.Centre, Hôpital Necker-Enfants Malades, Paris, France; 10 Institut Curie, Genetics Department & INSERM U1339, Paris Cité University, France; 11 Institut Curie, SIREDO Oncology Center, PSL Research University, Paris, France

**Keywords:** Mosaicism, Tetrasomy 18, Hepatoblastoma, Predisposition, Tumor evolution

## Abstract

**Background & Aims:**

Constitutive chromosomal abnormalities often underlie developmental defects, while somatic copy-number alterations are recurrent events in cancers. Although these processes are usually studied separately, they may occasionally converge, as illustrated by the increased incidence of hepatoblastoma in children with trisomy 18. Mosaic chromosomal abnormalities, present in only a fraction of cells, offer a unique biological context to explore how developmental defects and tumorigenesis can coexist within the same individual.

**Methods:**

We employed an integrated genomic approach, combining fluorescent *in situ* hybridization (FISH), whole genome sequencing (WGS), and single-nucleus RNAsequencing (snRNAseq) to characterize molecular alterations in a female patient presenting with multiple developmental delays and hepatoblastoma diagnosed at age two.

**Results:**

We identified a mosaic supernumerary derivative chromosome 18, karyotypically described as 47,XX,+der(18), in 10% of the patient’s liver and blood cells and in 100% of tumor cells. Integrated WGS and FISH analyses revealed that the der(18) resulted from complex chromosomal rearrangements involving eight breakpoints spanning 18p11.32 to 18q12.1. Haplotype phasing indicated a pre-zygotic origin, with mosaicism resulting from early embryonic rescue via loss of the der(18). SnRNAseq of liver and hepatoblastoma tissues enabled reconstruction of copy-number evolution across development and tumorigenesis and supported the prioritization of a restricted set of dosage-sensitive chromosome 18 candidate genes recurrently deregulated in hepatoblastoma. The patient has remained recurrence-free for 32 months following surgical resection and chemotherapy.

**Conclusions:**

This case reconstructs the molecular evolution of chromosome 18 alterations from the zygote to tumor formation and highlights how mosaic chromosome abnormalities can intersect with developmental defects and pediatric liver cancer.

**Impact and implications:**

This case study illustrates how a single chromosomal abnormality can influence both human development and cancer formation. By combining genomics and single-cell analyses, we traced the history of a rare chromosome 18 rearrangement from its origin before fertilization to its role in hepatoblastoma, a childhood liver cancer. We found that this abnormal chromosome was present in only a small fraction of normal liver and blood cells, but in all tumor cells, indicating that the cancer arose from one of these mosaic cells. Integrating single-cell and transcriptomic data enabled the prioritization of a restricted set of chromosome 18 candidate genes. This work provides a detailed example of how complex chromosomal alterations can emerge early in life, be selectively lost during development, and still leave a small population of abnormal cells that may later give rise to cancer. These findings highlight the need to consider mosaic chromosomal abnormalities as possible predisposing factors even in patients without obvious genetic syndromes, and they show the value of combining cytogenetic, genomic, and single-cell approaches to understand how cancer can develop from early developmental events.

## Highlights

• A child with developmental defects and hepatoblastoma carried a mosaic supernumerary derivative chromosome 18.
• Complex chromosome 18 rearrangement with eight breakpoints caused partial tetrasomy.
• The rearrangement arose before fertilization but was partially lost during development.
• Mosaic cells persisted in multiple lineages and gave rise to the tumor clone.
• Multi-omics reconstruction revealed chromosome 18 evolution from zygote to cancer.

## Graphical abstract

**Figure:**
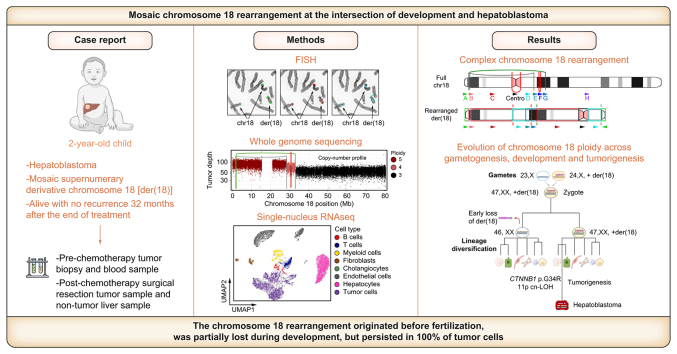

## Introduction

Chromosomal dosage imbalances, whether constitutional, mosaic, or somatic, can profoundly influence human development and contribute to cancer formation. Among them, constitutional abnormalities involving chromosome 18 cause a spectrum of developmental disorders. Trisomy 18 (Edwards syndrome), caused by the presence of an additional full chromosome 18, is the second most frequent autosomal trisomy syndrome after trisomy 21. It is associated with severe congenital anomalies and a 1-year survival rate of only ∼15%.^1^ In contrast, tetrasomy 18p – typically resulting from an isochromosome 18p alongside two normal chromosome 18 copies – is far rarer but associated with milder clinical features and near-normal life expectancy in many cases.^2^ In addition to these well-characterized syndromes, more complex abnormalities can also occur, including supernumerary derivative chromosomes resulting from structural rearrangements. In most cases of trisomy 18 and tetrasomy 18p, the extra chromosome arises from nondisjunction during meiosis II, a pre-zygotic event, and is therefore present in all cells.^3^^,^^4^ However, rare cases of mosaicism, where only a fraction of cells contain the extra chromosome, occur in approximately 5% of trisomy 18 and 2% of tetrasomy 18p cases.^2^ Mosaics usually arise from a post-zygotic event, with the timing of this event influencing the extent of mosaicism across different organs and the severity of the resulting phenotype.

Beyond developmental defects, somatic copy-number variations are a hallmark of cancers. Aneuploidy in cancers is both a consequence of widespread chromosomal instability and a driving force in cancer development and progression when it affects cancer driver genes.^5^^,^^6^ Somatic copy-number alterations can serve as preneoplastic precursor events when they originate in non-tumor cells.^7^ During clonal evolution, they accumulate in cancer cells, either as discrete events or as part of complex events such as chromothripsis. Chromosome missegregation can lead to chromosome-wide gains or losses, while structural variations, also known as chromosomal rearrangements, can induce focal copy-number variations.^8^^,^^9^ Most structural variations are combinations of simple events, classified as deletions, insertions, inversions, duplications, or translocations, with breakpoints detectable through short-read sequencing.^10^

Some aneuploidy syndromes are associated with an increased risk of developing certain types of cancer. Edwards syndrome is known to predispose mainly to hepatoblastoma (HB) and nephroblastoma, even though the molecular mechanisms are still unknown.^1^^,^^11^^,^^12^ In contrast, no cancer cases have been reported in tetrasomy 18p patients, suggesting that genes linked to HB development in Edwards syndrome reside on chromosome 18q.^2^

HB is a rare malignant tumor, accounting for 0.5-2% of pediatric malignancies, yet it is the most frequent liver cancer in childhood, with a median age of onset of 18 months. Classic risk factors include prematurity and low birth weight.^13^ While HB is mostly sporadic and non-syndromic, it is occasionally associated with genetic syndromes such as familial adenomatous polyposis, Beckwith-Wiedemann syndrome, Simpson-Golabi-Behmel syndrome, and Edwards syndrome.^14^ Recent research suggests that genetic predisposition may be more common than previously thought. For instance, Beckwith-Wiedemann syndrome, typically caused by germline or mosaic alterations of the 11p15.5 locus, accounts for only 2% of HB cases. However, 11p15.5 mosaic alterations have been identified in the liver of nearly 20% of patients with HB. These liver-specific alterations, likely resulting from a late post-zygotic event in hepatobiliary progenitors, are preneoplastic since the same 11p15.5 alteration is consistently found in the associated HB.^15^ Mosaicism has also been reported in patients with HB and mosaic Edwards syndrome^12^^,^^16^^,^^17^ and mosaic trisomy 7.^18^ Interestingly, in one case, the tumor cells did not harbor trisomy 18, suggesting a non-cell autonomous effect of the extra chromosome 18 on cancer predisposition.^19^

Here, we report a child with a mosaic supernumerary derivative chromosome 18 leading to atypical partial tetrasomy 18, who was diagnosed with HB at age two. Through combined analyses of whole-genome sequencing (WGS) and fluorescence *in situ* hybridization (FISH), we resolved the genetic events leading to the chromosomal aberrations. Our findings revealed that the supernumerary chromosome is not an isochromosome 18p but instead results from a complex rearrangement involving eight different breakpoints. Finally, by integrating WGS and single-nucleus RNAseq, we reconstructed the evolution of chromosome 18 alterations through gametogenesis, embryonic development, and tumorigenesis.

## Materials and methods

### Clinical samples

Written informed consent of the parents was obtained in accordance with French legislation. The study protocol was approved by the local Ethics Committee (CCPRB Paris Saint-Louis). We collected clinical, radiological and biological data of the patient. All tumor samples were immediately frozen in liquid nitrogen and stored at −80 °C. Four samples from two different timepoints were analyzed by next-generation sequencing (NGS): first, a pre-chemotherapy tumor biopsy (#06646T) and a blood sample (#06646S) taken at the time of tumor diagnosis; then, a tumor sample (#06312T) and a non-tumor liver sample (#06311N) from the surgical resection after neoadjuvant chemotherapy. Blood samples collected at diagnosis were used for molecular cytogenetics.

### Molecular cytogenetics

Molecular cytogenetics analyses were performed using the Agilent CGH Microarray 60 K (Agilent Technologies, Santa Clara, CA, USA) on circulating lymphocytes for genomic copy number analyses according to the manufacturer’s recommendations. Agilent CytoGenomics v5.0.2 software was used to analyze and report the data. Genomic positions are relative to human genome Build GRCh37/hg19. Using standard protocols, chromosomal rearrangement characterization and parental testing were performed by FISH with chromosome 18 region-specific probes on chromosome preparations and interphase nuclei from the patient’s leukocyte cultures (chromosome 7 centromeric and telomeric probes were also used as controls). The chromosome 18 probes used were 18ptel, 18qtel, CTD-2210P6 (18p11.32), RP11-694O17 (18p11.31), RP11-679K2 (18q11.2), and RP11-474H13 (18q12.1).

### Next-generation sequencing (NGS)

The biopsy was first analyzed by a custom NGS panel known as DRAGON (Detection of Relevant Alterations in Genes involved in Oncogenetics by NGS), commercially available as SureSelect CD Curie CGP by Agilent, targeting 571 genes of interest in oncology and an additional backbone of probes every 20 kb across the whole genome. The diagnostic biopsy was also sequenced as part of the PFMG2025 (Plan France Médecine Génomique) program on the SEQOIA platform, using blood as a non-tumor control. For these approaches, DNA and RNA were extracted according to standardized protocols as described previously.^20^ The PFMG2025 has established high-throughput genome sequencing (including paired germline/tumor WGS 60x, whole-exome sequencing 150x, and RNAseq) within standard of care, including rare tumors at diagnosis. The resected tumor was sequenced as part of the GePeLin research program, using non-tumor liver as a control. Briefly, we extracted DNA and RNA with the AllPrep DNA/RNA/miRNA Universal Kit (Qiagen), WGS (60X for the tumor and 30X for the non-tumor liver) and RNAseq were performed by Macrogen (Amsterdam) using NovaSeq (Illumina). WGS and RNAseq raw data were aligned on hg38 genome and variant called with the Dragen software (Illumina).

### Copy-number analysis and structural variants from NGS results

Copy-number profiles from the DRAGON targeted panel were estimated using the Facets package (v0.6.0)^21^ with a sex-specific unmatched-germline control previously sequenced using the same panel for normalization. For WGS, we also used the Facets tool on tumor and matched non-tumor as in.^22^ Structural variants were detected in WGS with the Manta tool.^10^

### Single-nucleus RNAseq

Nuclei were isolated from two fresh-frozen samples (tumor #06312T, non-tumor liver #06311N) using the Nuclei Isolation with RNase Inhibitor Kit (10X Genomics), and immediately fixated with the Evercode Nuclei Fixation v3 (Parse Biosciences). Single nuclei library preparation was performed using the Evercode WT v3 kit (Parse Biosciences) and sequenced by Macrogen (Amsterdam). Alignment and counting were performed with the Trailmaker platform, which uses the barcode rank plot to distinguish cell barcodes from background, leaving 6,380 cells for sample #06311N and 12,624 cells for sample #06312T. Then, an automatic cell type annotation was performed using the Liver Cell Atlas dataset as a reference,^23^ followed by manual validation. Subsequently, a quality control based on each cluster’s expression and size reduced the dataset to 5,706 cells for sample #06311N and 10,228 cells for sample #06312T. We used the InferCNV tool (https://github.com/broadinstitute/inferCNV) to reconstruct copy-number alterations using gene expression at the single-cell level. Differentially expressed genes were defined using an adjusted *p* value <0.05 and an absolute log_2_ fold change >0.25, a commonly used threshold in single-cell and bulk transcriptomic analyses to balance sensitivity and biological relevance. Rank-based enrichment analyses were additionally performed to ensure that results were not driven by arbitrary expression thresholds.

## Results

### Clinical report

The propositus (index case) is a four-year-old female, the only child of unrelated healthy parents of French origin. Her mother had two miscarriages, and her father was adopted with unknown medical background. Fetal ultrasounds during pregnancy revealed a single umbilical artery, intrauterine growth restriction, and a right pelvic kidney. She was born at 41 weeks of gestation after vaginal delivery with the following birth parameters: weight 2,660 g (1^st^ percentile), size 48 cm (9^th^ percentile), head circumference 32 cm (2^nd^ percentile). She presented with feeding difficulties, failure to thrive, microcephaly, and morphological features with thin upper lip, dysplastic ears, and bilateral partial 2/3 toes syndactyly. Her psychomotor milestones were delayed with hypotonia (sitting at 9-10 months, walking unaided at 26 months, first words around 24 months). She had moderate left-sided hearing loss. Cardiac ultrasound was normal. At 23 months, she had vomiting and constipation for a few weeks. Concurrently, the family consulted a pediatric neurologist regarding developmental delay. Abdominal palpation during clinical examination detected a liver mass. A thoracic-abdominal-pelvic computed tomography scan confirmed the presence of a large hepatic mass of 12 × 9 × 14 cm, involving segments IV, V, VII, VIII and upper VI. A biopsy revealed an epithelial HB of embryonal type, and no hepatic disease was identified in the non-tumor liver. The HB was classified as PRETEXT III with no metastasis. Alpha-fetoprotein (AFP) serum levels were 1,360,000 ng/ml. The child was included in the current international protocol PHITT and HEPATOBIO database with the parents’ informed consent. The tumor was classified as low-risk PHITT, B group.

Due to the association of pediatric malignancy, malformations, growth delay, and microcephaly, Fanconi anemia was initially suspected. Given the urgency to initiate treatment under the suspicion of Fanconi anemia, a course of vincristine and Irinotecan was begun, considering that cisplatin may be hazardous in the context of a DNA repair disorder, as cisplatin directly targets DNA. Subsequently, Fanconi anemia was ruled out via a chromosomal fragility test. The patient then received two courses of cisplatin at a dose of 2.7 mg/kg/day. Following the three chemotherapy courses, the tumor size reduced to 7 × 5 × 8 cm, and AFP levels dropped to 123,459 ng/ml. The patient underwent a right liver lobectomy and partial resection of the first segment. During surgery, the surgeon identified and resected a Meckel’s diverticulum and a urachal remnant. Histopathologic examination of the tumor revealed a mixed HB, with fibrous and necrotic alterations accounting for 65% of the lesion volume. The residual viable tumor predominantly exhibited classical fetal components, with minor embryonal, mesenchymal, and cholangioblastic components (Fig. S1). The pathologist confirmed the diagnoses of Meckel’s diverticulum and urachal remnant. Finally, the patient received four additional courses of cisplatin. Upon completion of treatment, AFP levels further decreased to <2 ng/ml. Currently, she remains recurrence free 32 months after treatment. She is gradually catching up on her psychomotor and growth delay, with her weight at -1 standard deviation and her height within the average range.

### Molecular characteristics of the tumor

Tumor sequencing was first performed on the pre-chemotherapy biopsy (sample #06646T) as part of routine molecular analysis for HB through a targeted NGS panel (DRAGON) for preliminary assessment and through WGS as part of the French national genomics program (PFMG2025, SeqOIA platform). A post-chemotherapy sample from the resection (sample #06312T) was later sequenced in WGS as part of the GePeLin research program. All platforms identified a somatic hotspot *CTNNB1* mutation (chr3:41224612G>C, p.G34R) leading to β-catenin activation as well as a copy-neutral loss of heterozygosity of the 11p chromosome (encompassing the 11p15.5 imprinted locus), which are the two most prevalent driver alterations of HB.^24^ There was no evidence of 11p15.5 mosaicism in the non-tumor liver sample analyzed. We also identified a few copy-number alterations shared by the pre-chemotherapy biopsy (#06646T) and the post-chemotherapy resection (#06312T), including a loss of 1p, a gain of 1q and alterations of chromosome 18 (Fig. S2). Additionally, the post-chemotherapy tumor exhibited numerous private copy-number alterations, as opposed to the biopsy which only had a private loss of chromosome 9, which may suggest an impact of cisplatin or irinotecan on chromosomal instability (Fig. S2). Notably, in both tumor samples, chromosome 18 presented a complex and fragmented copy-number profile, ranging from 2 to 5 copies, which is unusual in HB.^24^

### Identification of a mosaic supernumerary chromosome 18 derivative

**Fig. 1:**
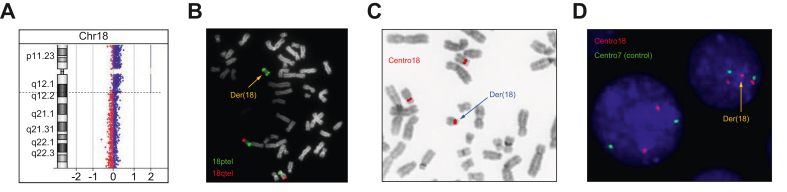
aCGH and FISH identify a mosaic chromosome 18 derivative. (A) aCGH profile of chromosome 18 showing a 27 Mb gain across 18p11.32–q12.1 in the patient’s leukocytes. (B-D) FISH with various chromosome 18 probes performed on circulating lymphocytes in metaphase (B,C) or interphase (D). (B) Probes for p and q telomeric regions reveal a rearranged chromosome 18 highlighted by the yellow arrow. (C) Centromeric probe showing the rearranged chromosome 18. (D) Interphase nuclei with the centromeric probe, revealing mosaicism with the coexistence of cells disomic (left) and trisomic (right) for chromosome 18. aCGH, array comparative genomic hybridization; der(18), derivative chromosome 18; FISH, fluorescent *in situ* hybridization.

Based on the clinical profile and the identification of a chromosome 18 rearrangement in the tumor, we investigated the possibility of a constitutional chromosome 18 rearrangement. We first performed array comparative genomic hybridization (aCGH) on the patient's leukocytes, which revealed a 27 Mb mosaic gain involving the 18p11.32-q12.1 region: arr[GRCh37] 18p11.32q12.1(14316-27890123)x2∼3 (Fig. 1A). Subsequently, FISH analysis was conducted on cultured lymphocytes from the patient and her parents, using chromosome 18-specific telomeric and centromeric probes. This analysis showed no abnormalities in the parents' lymphocytes but identified a *de novo* rearranged chromosome 18 (der(18)) in 8% of the patient’s cells, showing a p-telomeric probe signal at both ends (Fig. 1B-D). These findings were initially interpreted as a derivative of a chromosome 18 pericentric inversion, der(18)inv(18)(p11.21q12.1). The der(18) was present in addition to the two normal copies of chromosome 18 in 8% of leukocytes (47,XX,+der(18)), while the other 92% were diploid for chromosome 18 (46,XX), indicative of a mosaic alteration (Fig. 1D).

**Fig. 2:**
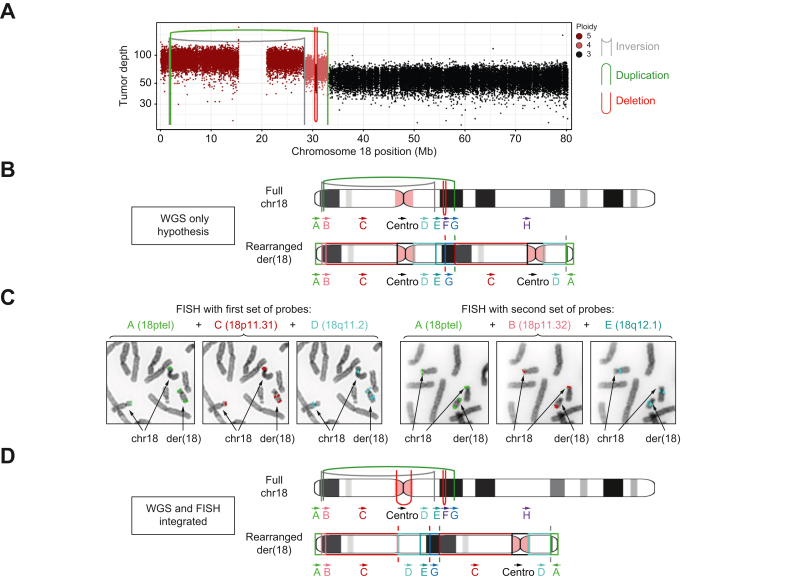
Complex rearrangements of chromosome 18 resolved through WGS and specific FISH. (A) Sequencing depth along chromosome 18 in the tumor sample #06312T, colored by inferred ploidy in the tumor. The structural variants are shown in green (duplication), red (deletion), and grey (inversion). (B) Chromosome 18 rearrangement hypothesized from WGS showing regions (A to H) delimited by the breakpoints and used to design five FISH probes. (C) FISH results with probes A, C and D (left) and A, B and E (right), validating the hypothesized rearranged der(18) with an extra deletion encompassing the first centromere as detailed in (D). der(18), derivative chromosome 18; FISH, fluorescent *in situ* hybridization; WGS, whole-genome sequencing.

Given the chromosome 18 abnormalities detected by WGS in the tumor, we hypothesized that the rearranged der(18) chromosome observed in mosaic form in lymphocytes was also retrieved in the HB. However, the copy-number profile of chromosome 18 in the tumor showed greater complexity than expected for an inv(18)(p11.21q12.1) (Fig. 2A), prompting us to investigate in detail the structural variants from WGS data. In the tumor, we identified three structural variants: an inversion (chr18:1,781,448–28,388,920), a 500 kb deletion (chr18:30,373,049–30,869,237) and a 21 Mb duplication (chr18:1,983,741–32,966,268) (Fig. 2A). The six breakpoints from the three structural variants explained the alternating copy-number profile of the tumor. Together with the centromere, they defined eight genomic regions on chromosome 18 as indicated in Fig. 2B: Pentasomy of region A (1.8 Mb), [GRCh38]18p11.32(1–1,781,448); Tetrasomy of region B (0.2 Mb), [GRCh38]18p11.32(1,781,449–1,983,741); Pentasomy of region C (13.9 Mb), [GRCh38]18p11.32p11.1(1,983,742–15,948,602); Pentasomy of region D (7.5 Mb), [GRCh38]18p11.1q12.1(20,914,128–28,388,920); Tetrasomy of region E (2 Mb), [GRCh38]18q12.1(28,388,921–30,373,049); Trisomy of region F (0.5 Mb), [GRCh38]18q21.1(30,373,050–30,869,237); Tetrasomy of region G (2 Mb), [GRCh38]18q21.1(30,869,238–32,966,268); Trisomy of region H (47 Mb), [GRCh38]18q21.1q23(32,966,269–80,373,285).

This copy-number profile suggested that tumor cells carried three copies of the full chromosome 18 in addition to the rearranged der(18), leading to pentasomy in regions A, C and D. Based on these structural variants, we were able to hypothesize the complex structure of the rearranged der(18) presented in Fig. 2B. The hypothesized der(18) was consistent with the p-telomeric probe signal seen at both ends in FISH (Fig. 1B). However, WGS predicted a rearranged der(18) with two centromeres (Fig. 2B), which contradicted the FISH results showing a single centromere (Fig. 1C,D). To reconcile these two findings, we performed additional FISH experiments with five probes spanning regions A to D, to validate the reconstruction from WGS (Fig. 2B). Using two sets of three probes on the same lymphocytes, we confirmed the segmental organization predicted by the structural variants detected in WGS (Fig. 2C), except for the presence of only one chromosome 18 centromere. This validated our hypothesis that the same rearranged chromosome was found in mosaic in leukocytes and clonally in the tumor. The presence of a single centromere in FISH (Fig. 1C,D) suggested that there was a fourth structural variant event, not detected by WGS, leading to the loss of one of the centromeres. The probable breakpoints of this deletion would be in the centromeric region, which is not covered in short-read WGS. The localization of the two FISH probe C signals on the long arm of the rearranged chromosome 18, together with the observation of FISH probe D signals on both arms (Fig. 2C), allowed us to identify which centromere had been lost during the series of complex events leading to the rearrangement of chromosome 18, as schematized in Fig. 2D.

Overall, WGS and targeted FISH revealed a more complex chromosome 18 rearrangement than initially suggested by aCGH and FISH. Instead of a simple der(18)inv(18)(p11.21q12.1), the analysis revealed multiple structural variants involving at least six and hypothetically eight DNA breakpoints, leading to variable copy-number changes between 18p11.32 and 18q12.1 (Fig. 2). Notably, mosaic blood cells exhibited alternating tetrasomy, trisomy, and disomy along chromosome 18, while the tumor showed an extra copy of a full chromosome 18, resulting in pentasomy in some regions (Fig. 2 and S3). In summary, 8% of the patient's lymphocytes carried a derivative of a complex rearrangement of chromosome 18, generating genomic imbalances in the short arm and long arm regions. In tumor cells, this marker was present in all cells. This is the first case of partial genomic imbalance involving the long arm of chromosome 18 associated with HB.

### Investigation of the mosaic der(18) in the non-tumor liver

Given that FISH identified the rearranged chromosome 18 as mosaic in 8% of blood cells and WGS retrieved the same der(18) in all tumor cells, we wanted to investigate whether the der(18) was present in the non-tumor liver cells. We first analyzed RNAseq data and compared the mean expression of coding genes on chromosome 18p in the non-tumor liver of the patient, which appeared to be highly upregulated compared to 33 non-tumor livers of other children (Fig. S4A). Then, we assessed allelic imbalance in WGS data from the non-tumor liver by examining B-allele frequency informed by the tumor, and found a clear disequilibrium in the rearranged region (0 to 33 Mb) of chromosome 18 (Fig. S4B). The chromosome 18 breakpoints first identified in the tumor were also retrieved in the non-tumor liver, but with an allele frequency around 10 times lower. Sequencing depth in segments defined by these breakpoints also showed variations but with lower amplitude than in the tumor (Fig. S4C). These results indicate that the rearranged chromosome 18 identified in the tumor was also present in a fraction of the non-tumor liver cells.

**Fig. 3:**
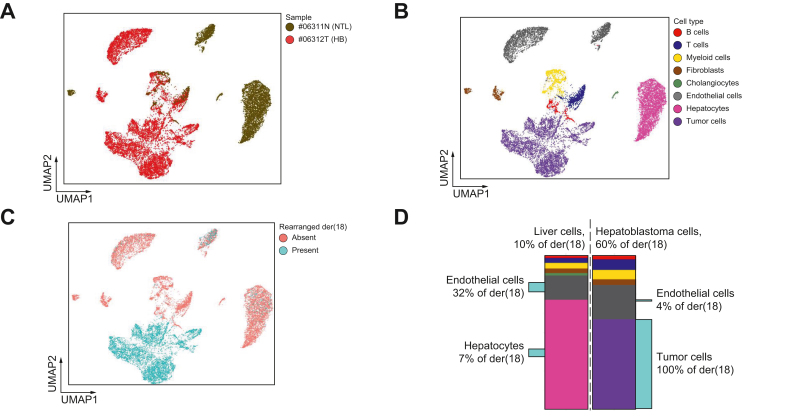
Single-cell analysis of the rearranged chromosome 18 in liver and hepatoblastoma. UMAPs showing, for each cell, the sample of origin (A), the cell type (B), or the presence of the rearranged chromosome 18 (C). (D) Proportion of cell types in the two samples, with the relative number of cells harboring the rearranged chromosome 18 in each cell type indicated. der(18), derivative chromosome 18; HB, hepatoblastoma; NTL, non-tumor liver; UMAP, uniform manifold approximation and projection.

To further identify which cells in the liver harbored the rearranged chromosome der(18), we performed single-nucleus RNAseq (snRNAseq) on the same post-chemotherapy non-tumor liver (#06311N) and HB (#06312T) samples originally sequenced in WGS. We identified a total of 15,934 cells from the two samples and characterized their cell types (Fig. 3A,B). Using the InferCNV tool to reconstruct copy-number alterations from RNA expression at the single-cell level, we identified in all tumor cells the same alterations found in WGS, including gains of chr1q, 8, 18p and 20 (Fig. S5). As suggested by previous bulk RNAseq and WGS results, chromosome 18 imbalance was also detected in a subset of non-tumor liver cells: 7% of hepatocytes and 32% of endothelial cells harbored the alteration, corresponding to an overall liver mosaicism of 10% (Fig. 3C,D). In the HB sample, in addition to tumor cells, 4% of endothelial cells also carried the rearranged der(18), indicating that angiogenesis recruited both diploid and mosaic endothelial cells into the tumor (Fig. 3C,D). Finally, the alteration was not detected in any other cell types, including T cells, B cells, and myeloid cells, in contrast to blood samples, in which FISH identified der(18) in 8% of leukocytes. Overall, this experiment confirmed the mosaicism in the non-tumor liver suggested by the bulk analysis, and highlighted the variable prevalence of the der(18) among different cells. It is noteworthy that the rearranged chromosome 18 was found clonally in the tumor, indicating that it was present in its cell of origin, while it was only found in 7% of non-tumor hepatocytes.

### Timing and evolution of chromosome 18 alterations

**Fig. 4:**
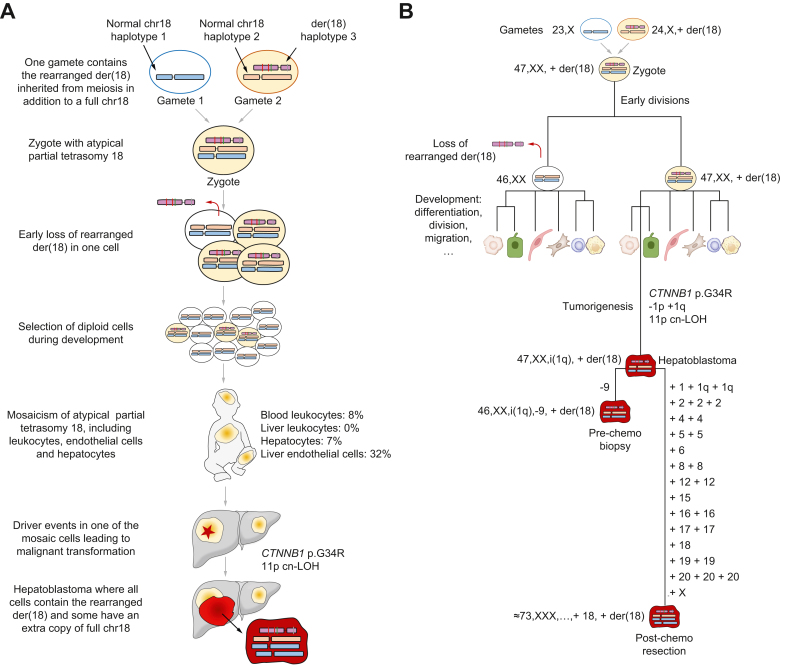
Evolution of chromosome 18 ploidy during gametogenesis, development and tumorigenesis. The natural history of the patient (A) and the phylogenetic evolution focused on copy-number alterations (B) are presented from gametogenesis to tumorigenesis by combining insights from FISH, WGS, and snRNAseq. The presence of three different haplotypes found in WGS indicates that the rearrangement of chromosome 18 was a pre-zygotic event. The low-level mosaicism observed in FISH, WGS and snRNAseq indicates that the der(18) was lost in a fraction of cells during early development and then counter-selected. The analysis of copy-number alterations in the pre- and post-chemotherapy tumor samples by WGS enabled reconstruction of the patient’s copy-number phylogeny. der(18), derivative chromosome 18; FISH, fluorescent *in situ* hybridization; snRNAseq, single-nucleus RNA sequencing; WGS, whole-genome sequencing.

Finally, we investigated the timing of acquisition of the rearranged chromosome 18. Mosaicism is usually indicative of a post-zygotic event, explaining why only a fraction of cells harbor the alteration. However, the patterning of B-allele frequency along chromosome 18 in the non-tumor liver was unusual (Fig. S6A) compared to other mosaic copy-number alterations,^15^ suggesting the possibility that three different haplotypes of chromosome 18 co-existed in the patient’s DNA. To validate this hypothesis, we queried a list of multiallelic single nucleotide polymorphisms retrieved from Phillips *et al.*^25^ to investigate the potential co-existence of three different alleles. Indeed, we detected three concomitant alleles in six loci among 40 multiallelic SNPs present in the regions of the rearranged chromosome 18 (Fig. S6B). The presence of these three haplotypes suggests that one of the two parents contributed to two different haplotypes of chromosome 18, presumably through one normal copy and the rearranged chromosome. As presented in Fig. 4A, this implies that the rearranged chromosome 18 originated from a parent meiosis division. This contradicts the hypothesis that the der(18) occurred post-zygotically. Instead, it suggests that the zygote already harbored the rearranged chromosome 18. In this context, the mosaicism should result from reversion through loss of der(18) by a cell during early development, and the low prevalence of mosaic cells (8% in blood, 10% in liver) suggests that there was a counter-selection of aneuploid cells or a selection of diploid cells during development (Fig. 4A). The presence of the same chromosome 18 alterations in both non-tumor and tumor samples indicates that the cell-of-origin of the tumor was part of the mosaic cells carrying the rearranged chromosome 18, and the final copy-number profile in the tumor is explained by an extra-gain of a full chromosome 18 in a subset of the tumor (Fig. 4A). Finally, by combining all information from FISH, WGS, and snRNAseq, including the different copy-number alterations found in the two tumor samples (Fig. S2), we were able to reconstruct a phylogenetic tree to recapitulate the evolution of ploidy during gametogenesis, development and tumorigenesis (Fig. 4B).

### Candidate genes linking chromosome 18 aberrations and HB

**Fig. 5:**
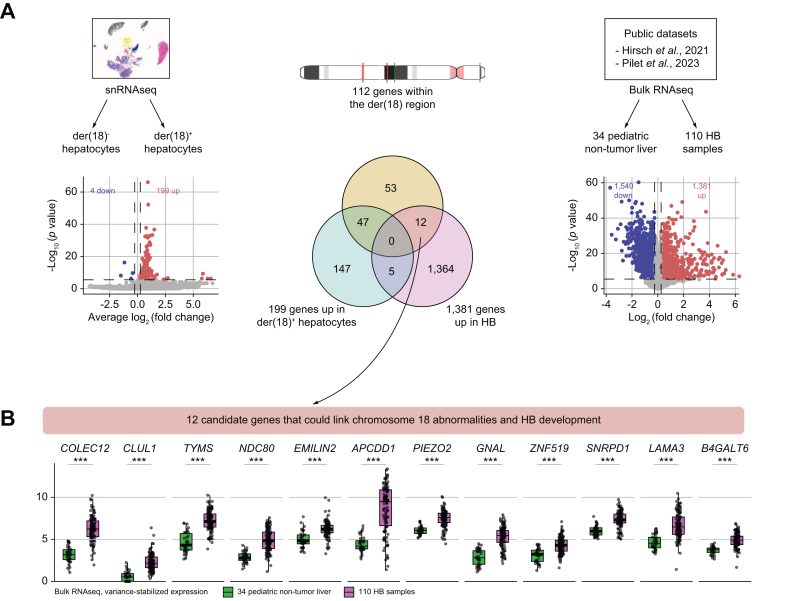
Identification of candidate genes linking chromosome 18 abnormalities and hepatoblastoma development. (A) Strategy to prioritize candidate genes within the rearranged chromosome der(18). Differential expression analyses were performed between der(18)+ and der(18)- hepatocytes using snRNAseq (left), and between HB and pediatric non-tumor liver samples using bulk RNAseq from public datasets^15^^,^^24^ (right). The Venn diagram shows the overlap between genes located in the der(18) region (112 genes), genes upregulated in der(18)+ hepatocytes, and genes upregulated in HB (adjusted *p* value <0.05 and |log_2_ fold-change| >0.25). (B) Expression of 12 candidate genes in bulk RNAseq data from pediatric non-tumor liver (green, n = 34) and HB samples (pink, n = 110), shown as variance-stabilized expression values. Statistical significance was assessed using a two-sided Wilcoxon rank-sum test (∗∗∗*p* <0.001). der(18), rearranged chromosome 18; HB, hepatoblastoma; snRNAseq, single-nucleus RNA sequencing.

Although the association between trisomy 18 and HB has been reported in more than 70 cases, no specific locus on chromosome 18 has been definitively implicated in HB development to date.^11^^,^^12^ Because the present case represents the first HB associated with a partial chromosome 18 anomaly, it provides an opportunity to refine the genomic regions potentially involved in HB predisposition. Among the 266 protein-coding genes located on chromosome 18 (GENCODE release 43), 112 map to the duplicated segments of the der(18) chromosome, including 95 genes present in tetrasomy and 17 in trisomy (Fig. 5A, Table S1).

To prioritize candidate genes, we leveraged snRNAseq data and compared mosaic hepatocytes carrying the der(18) with diploid hepatocytes. Among 199 genes upregulated in der(18)+ hepatocytes, 47 were located within the der(18) region, representing a significant enrichment consistent with cis dosage effects (Fisher’s exact test *p* <0.001; GSEA rank test, adjusted *p* <0.001) (Fig. 5A, Table S1).

We next assessed deregulation of der(18) genes in HB using bulk RNAseq data from pediatric non-tumor liver (n = 34) and HB samples (n = 110). Among 1,381 genes upregulated in HB, only 12 mapped to the der(18) region, corresponding to a modest and non-significant enrichment (Fig. 5A, Table S1). Nevertheless, all 12 genes showed robust overexpression in HB samples (Fig. 5B), identifying them as candidate dosage-sensitive genes potentially linking chromosome 18 abnormalities and HB. Notably, this list includes *TYMS*, previously proposed to modulate treatment response in trisomy 18–associated HB.^16^

In contrast, none of these 12 genes overlapped with those upregulated in der(18)+ hepatocytes, suggesting that the transcriptional consequences of chromosome 18 gain may depend on developmental context or cell identity, consistent with a hepatoblast rather than mature hepatocyte cell-of-origin. Together, these analyses define a restricted set of chromosome 18 candidate genes recurrently deregulated in HB and provide a mechanistic framework for investigating how chromosome 18 dosage imbalance may contribute to HB predisposition.

## Discussion

In this report, we describe the first case of HB occurring in a patient with partial tetrasomy 18,^2^^,^^26^ associated with a complex rearrangement encompassing both the short arm and proximal long arm of chromosome 18. Trisomy 18 is known to predispose to HB, whereas classical tetrasomy 18p has not been associated with cancer, suggesting that chromosome 18 dosage imbalances may contribute to tumorigenesis in a region- and context-dependent manner.^1^^,^^2^^,^^11^^,^^12^ More generally, the question of the minimal region responsible for the Edwards syndrome phenotype, including HB predisposition, can be addressed by studying patients with partial trisomy or tetrasomy of chromosome 18^27^ and summarized in gene dosage maps.^28^^,^^29^ Despite more than 70 reported cases of HB in trisomy 18, no single chromosome 18 locus has been definitively implicated to date,^11^^,^^12^ although altered dosage of individual genes such as *TYMS* has been proposed to modulate treatment response.^16^

By integrating snRNAseq, bulk transcriptomic data from independent HB cohorts, and the precise mapping of duplicated segments on the der(18), we delineated a restricted set of candidate dosage-sensitive genes potentially linking chromosome 18 abnormalities and HB. Rather than identifying a single dominant driver locus, our analyses support a model in which multiple genes within the duplicated regions show modest but coherent deregulation in HB. The candidate genes did not converge on a single dominant biological pathway but instead spanned heterogeneous functions, supporting a cumulative dosage model rather than a single-gene mechanism. This observation is consistent with the polygenic nature of trisomy-associated phenotypes, in which cumulative dosage effects of many genes rather than a single causal gene contribute to disease risk, as exemplified in trisomy 21, where multiple dosage-sensitive genes distributed along chromosome 21 influence the phenotypic spectrum and partial trisomy studies have not isolated a single critical locus.^30^ Importantly, candidate gene prioritization revealed a clear dissociation between genes upregulated in der(18)+ hepatocytes and those overexpressed in bulk HB samples. This lack of overlap suggests that the transcriptional consequences of chromosome 18 gain depend on developmental stage, cellular identity, or tissue context. In particular, cis dosage effects observed in mosaic mature hepatocytes may differ from those operating earlier during development in hepatoblasts, which are thought to represent the hepatoblastoma cell of origin.^31^ These results also leave open the possibility that chromosome 18 abnormalities contribute to HB risk through non-cell-autonomous or field effects, rather than acting as direct tumor-maintenance drivers. In line with this possibility, differential expression analysis of endothelial cells bearing the der(18) identified several cis-upregulated genes within the duplicated chromosome 18 region, including *YES1* (data not shown), an oncogene previously implicated in endothelial cell migration.^32^ While no functional consequences can be inferred from the present data, this finding is compatible with subtle microenvironmental effects of chromosome 18 dosage imbalance in non-parenchymal liver cells.

More broadly, the mechanisms linking trisomy 18 and HB have so far remained elusive, with prevailing hypotheses favoring indirect effects related to abnormal growth and congenital malformations.^11^ Indeed, low birth weight, which is common in trisomy 18, is a risk factor for HB. Cases of HB in patients with Edwards syndrome could therefore be a consequence of this low birth weight. Congenital anomalies are also highly prevalent in both trisomy 18 and tetrasomy 18p, and are strongly associated with HB.^11^^,^^33^ In the present case, the presence of multiple embryonic remnants such as urachal remnant and Meckel’s diverticulum may suggest an incomplete regression of embryonic tissues, which might play a role in the emergence of embryonal tumors. However, urachal anomalies and Meckel’s diverticulum are usually associated with late malignant transformation in adults and not with embryonal tumors.^34^^,^^35^ Of note, cardiac malformations are the most common birth defects in both Edwards syndrome and tetrasomy 18p^2^^,^^11^ and a major contraindication to surgery in patients with HB and trisomy 18,^12^ but the present case exhibited no cardiac malformation, possibly because of the mosaicism.

A potential cell autonomous role of chromosome 18 alterations in HB development might also be envisioned: indeed, in the present case, the rearranged chromosome der(18) was only found in 10% of non-tumor liver cells, and most specifically in 7% of hepatocytes, while it was found in 100% of tumor cells, indicating that the tumor cell-of-origin harbored the der(18). This may suggest that the extra copies of chromosome 18 regions may have directly driven HB development and progression, particularly since an extra copy of a full chromosome 18 was also retrieved in the post-chemotherapy tumor sample. Nevertheless, a report of HB in a patient with mosaic Edwards syndrome, in which the tumor cells did not harbor trisomy 18,^19^ favors an indirect, non-cell autonomous role of the chromosome 18 gain. This is reinforced by the absence of chromosome 18 gains and driver alterations located on chromosome 18 in the vast majority of HB cases,^24^ which argues against a direct driving role of chromosome 18 in HB progression.

In the present case, the mosaic chromosome 18 derivative was minor (8-10%) but widespread (blood leukocytes, hepatocytes, endothelial cells, cells of mesenchymal and epithelial origin), explaining the patient’s various symptoms, which led to the identification of the genetic defect. It is nevertheless possible that other patients with restricted mosaicism of trisomy 18 or tetrasomy 18 remain underdiagnosed because of limited clinical symptoms. Thus, some HBs considered non-syndromic, and therefore not associated with a germline defect, could actually be related to mosaic trisomy or tetrasomy 18 without a clinical diagnosis, as previously shown for mosaic 11p15.5 alterations identified in nearly 20% of patients with HB despite the absence of a Beckwith–Wiedemann syndrome diagnosis.^15^ This raises the possibility that systematic analyses of tumor-adjacent non-tumor liver tissue, in addition to blood, may represent a valuable approach to uncover cryptic mosaic predisposition states that would otherwise remain undetected. Overall, estimating the true contribution of such mosaic alterations to HB risk will require a better understanding of their prevalence in the livers of the general population, which currently remains unknown because of limited access to healthy liver tissue.

Our integrated deep molecular investigation of the present case highlights both the limitations and the complementarity of FISH and WGS for the investigation of atypical chromosome 18 rearrangements and provides new insights into the mechanisms and timing of tetrasomy 18. Indeed, the detection of structural variants by WGS was necessary to resolve the complex chromosomal rearrangements involving eight DNA breakpoints, whereas the initial FISH experiment had identified only a simple inv(18)(p11.21q12.1). Nevertheless, one peri-centromeric deletion was undetectable by short-read WGS and was only recovered through FISH. The rearranged chromosome 18 resulting from this combination of duplications, inversions, and deletions presented an unusual copy-number profile, with regions of disomy, trisomy and tetrasomy between 18p11.32 and 18q12.1. This is reminiscent of the description of patients with tetrasomy 18p also presenting with trisomy in small regions of proximal 18q,^26^ which might harbor complex rearrangements instead of the suspected isochromosome 18p.

The haplotype analysis also revealed that the rearrangement of chromosome 18 in this patient was a pre-zygotic event, which is counter-intuitive since mosaicism is usually related to post-zygotic events. This supports the notion that mosaicism can arise from reversion of aneuploidy^36^ (Fig. 4). Interestingly, reversion of pre-zygotic aneuploidy can lead to the development of a fully euploid fetus when the aneuploid cells are segregated to the placental lineage.^37^ Here, the high percentage (>90%) of euploid cells in the blood and liver of the patient is indicative of a strong counter-selection of the aneuploid cells, since the reversion occurred at the earliest at the 2-cell stage, corresponding to 50% mosaicism in the absence of selection. Multiple independent reversion events might also have occurred throughout development. Importantly, the scenario of mosaicism induced by a pre-zygotic event followed by a reversion during development could explain other unusual presentations, such as a recent report of tetrasomy 18p mosaicism in two monozygotic twin sisters, which was hypothesized to result from a post-zygotic but pre-twinning event.^38^

In conclusion, complementary genetic analyses are necessary in patients with HB, especially with a background of malformations. Atypical partial tetrasomy 18 can be mistaken for an isochromosome 18p by standard FISH but the combination of FISH, WGS and snRNAseq can help to reconstruct the series of rearrangements and the evolution of chromosome 18 ploidy from gametogenesis to tumorigenesis.

## Abbreviations

aCGH, array comparative genomic hybridization; AFP, alpha-fetoprotein; FISH, fluorescent *in situ* hybridization; HB, hepatoblastoma; NGS, next-generation sequencing; PFMG2025, Plan France Médecine Génomique 2025; RNAseq, RNA sequencing; snRNAseq, single-nucleus RNA sequencing; WGS, whole genome sequencing.

## Authors’ contributions

E.C., M.C., Z.G., S.R., I.A., and T.Z.H. wrote the main manuscript text and figures. S.R., I.A., and T.Z.H. supervised the project. J.Z.R., S.R., I.A., and T.Z.H. designed the experiments. M.C., A.P., N.U., J.M.P., G.P., I.T., and S.R. performed the experiments. E.C., M.C., Z.G., A.P., G.M., G.S., I.T., D.S.L, J.Z.R., S.R., I.A., and T.Z.H. analyzed the data. G.M., F.S., A.G., C.Ch., C.Ca., G.S., J.M.P., and G.P. contributed materials. All authors reviewed the manuscript.

## Data availability

Raw sequencing data from whole genome sequencing and single-nucleus RNA sequencing experiments performed for this study have been deposited to the European Genome Archive (EGA) under accession code EGAS00001008072. Bulk RNAseq datasets from hepatoblastoma and non-tumor liver samples used for comparative analyses, generated previously by our group, are publicly available through the EGA under accession codes EGAS00001005108 and EGAS00001006692. These data contain identifiable genetic variants and are thus accessible under controlled access for patient privacy concerns by contacting the data access committee.

## Financial support

FunGeST team (FUNctional GEnomics of Solid Tumors) is supported by Ligue contre le cancer (équipe labellisée), SFCE (Société Française de Lutte Contre les Cancers et les Leucémies de l′Enfant), the SIRIC CARPEM, the SIRIC Paris Kids Cancer (PKC), PeLiCan. Resist InCa (Pediatric LIver CANcer database to combat RESISTance to treatment, Institut National du Cancer), France Génomique, CisMutHep InCa High-Risk High-Gain (Institut National du Cancer), the European Union under Grant Agreement Nr. 101136622 (THRIVE) and LabEx Immuno-Oncology, France 2030. MC was funded by the Fondation pour la Recherche Médicale (grant number FDM202206015442).

## Conflicts of interest

The authors declare no potential conflicts of interest.

Please refer to the accompanying ICMJE disclosure forms for further details.
