# Supplementary material for: Molecular evolution of mosaic chromosome 18 copy-number alterations from gametes to hepatoblastoma
Source: JHEP Rep. 2026 Apr 20;8(8):101862. doi: 10.1016/j.jhepr.2026.101862 (PMC13355473; doi:10.1016/j.jhepr.2026.101862)
Supplement: Multimedia component 1 [file mmc1.pdf]

Molecular evolution of mosaic chromosome 18 copy-number alterations from gametes to hepatoblastoma

Elise Cendres, Marianna Cornet, Zoé Gautier, Aurore Pire, Noémie Urvoy, Guillaume Morcrette, Fatoumata Simaga, Anne Guimier, Christophe Chardot, Carmen Capito, Gudrun Schleiermacher, Julien Masliah-Planchon, Gaelle Pierron, Ilaria Taddei, Dominique Stoppa-Lyonnet, Jessica Zucman-Rossi, Serge Romana, Isabelle Aerts, Theo Z Hirsch

Table of contents

Fig. S1……………………………………………………………………………………2

Fig. S2……………………………………………………………………………………3

Fig. S3……………………………………………………………………………………4

Fig. S4……………………………………………………………………………………5

Fig. S5……………………………………………………………………………………6

Fig. S6……………………………………………………………………………………7

Supplementary Figure 1

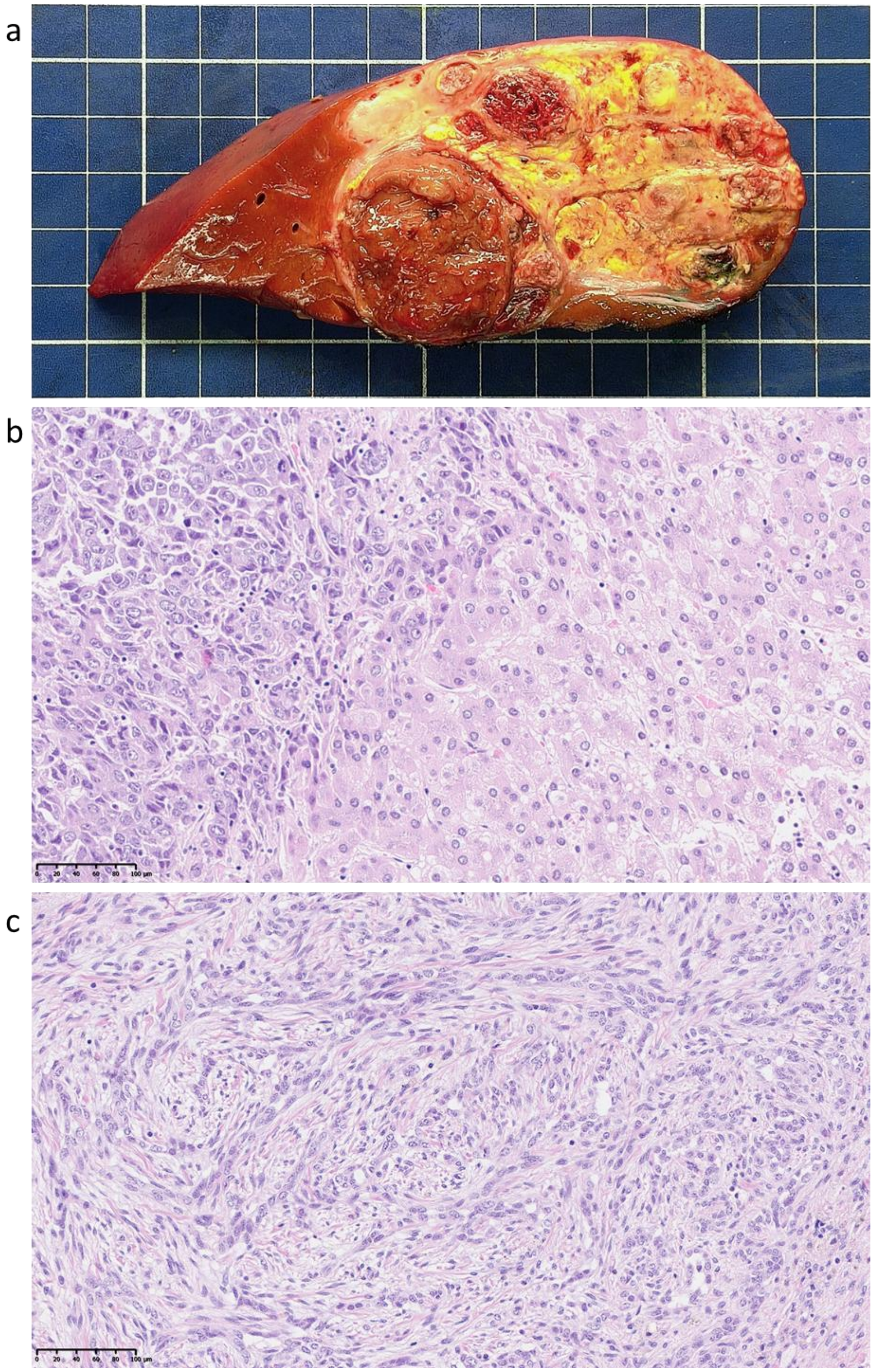

Fig. S1: Histological examination of the hepatectomy specimen

a) Hepatectomy specimen, gross examination: 8,5 cm x 5 cm neoplasm, rather well limited, partially encapsulated, with 65% fibro / necrotic changes. The non-tumor liver appears non cirrhotic. **b)** Hematoxylin Eosin x20: Hepatoblastoma, embryonal (left) and fetal (right) component. **c)** Hematoxylin Eosin x20: Hepatoblastoma, mesenchymal component.

Supplementary Figure 2

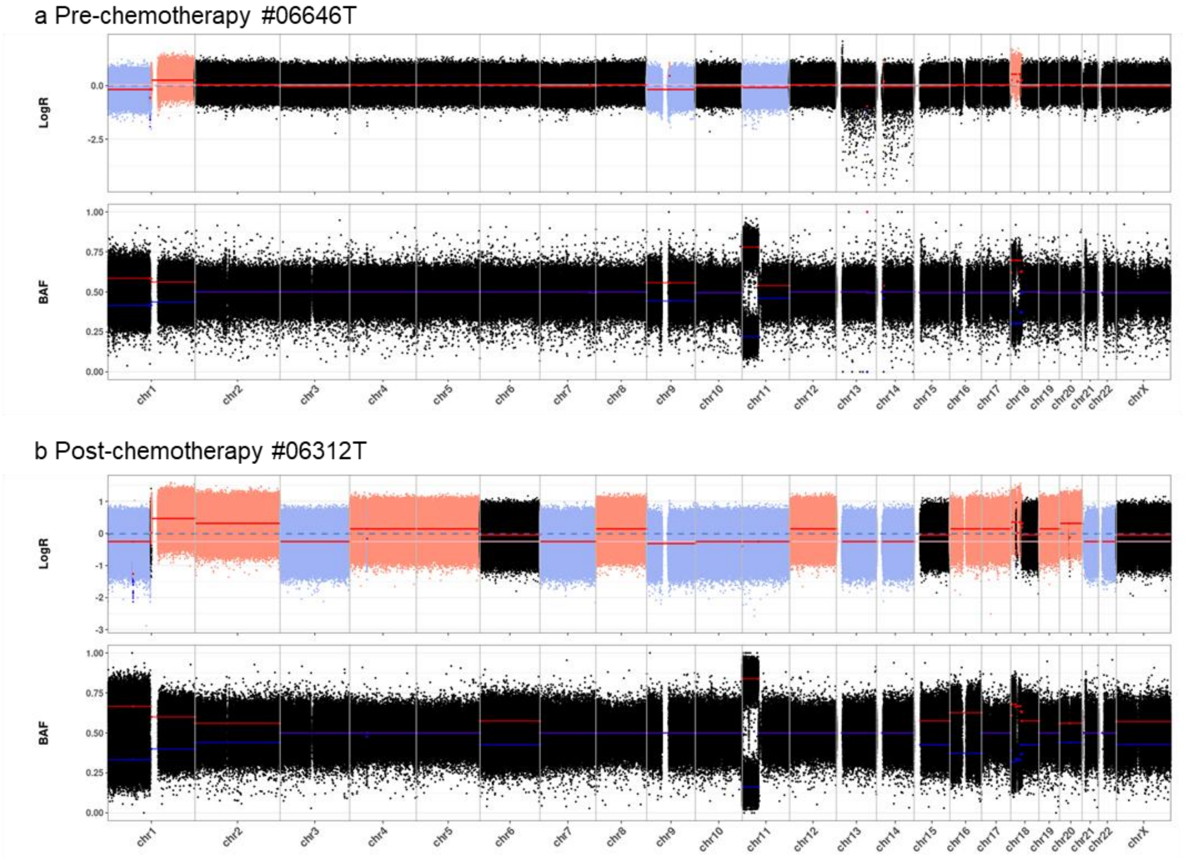

Fig. S2: Copy-number profile of the tumor samples

Copy-number profile of the tumor obtained from the pre-chemotherapy biopsy **(a)** and the post-chemotherapy resection **(b)**, constructed by the FACETS algorithm on Whole-Genome Sequencing data.

Supplementary Figure 3

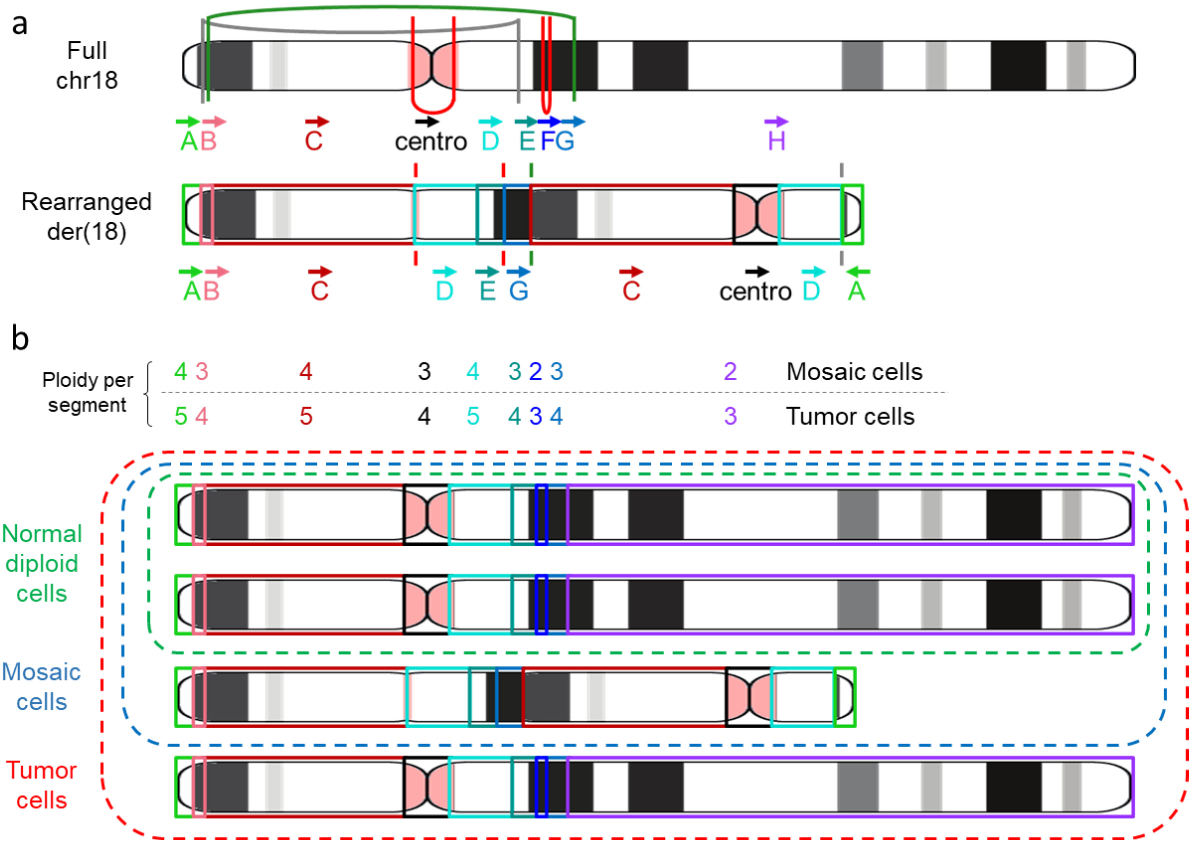

Fig. S3: Structural variants and their effect on chromosome 18 ploidy

a) Structural variants detected by Whole-Genome Sequencing and FISH explaining the rearrangement of the chr18. **b)** Explanation of how the rearranged der(18) leads to alternate tetrasomy, trisomy and disomy in mosaic cells. In tumor cells, an extra copy of a full chr18 adds 1 copy to each segment, giving copy numbers ranging from 3 to 5.

der(18): rearranged chromosome 18. FISH: Fluorescent in situ hybridization.

Supplementary Figure 4

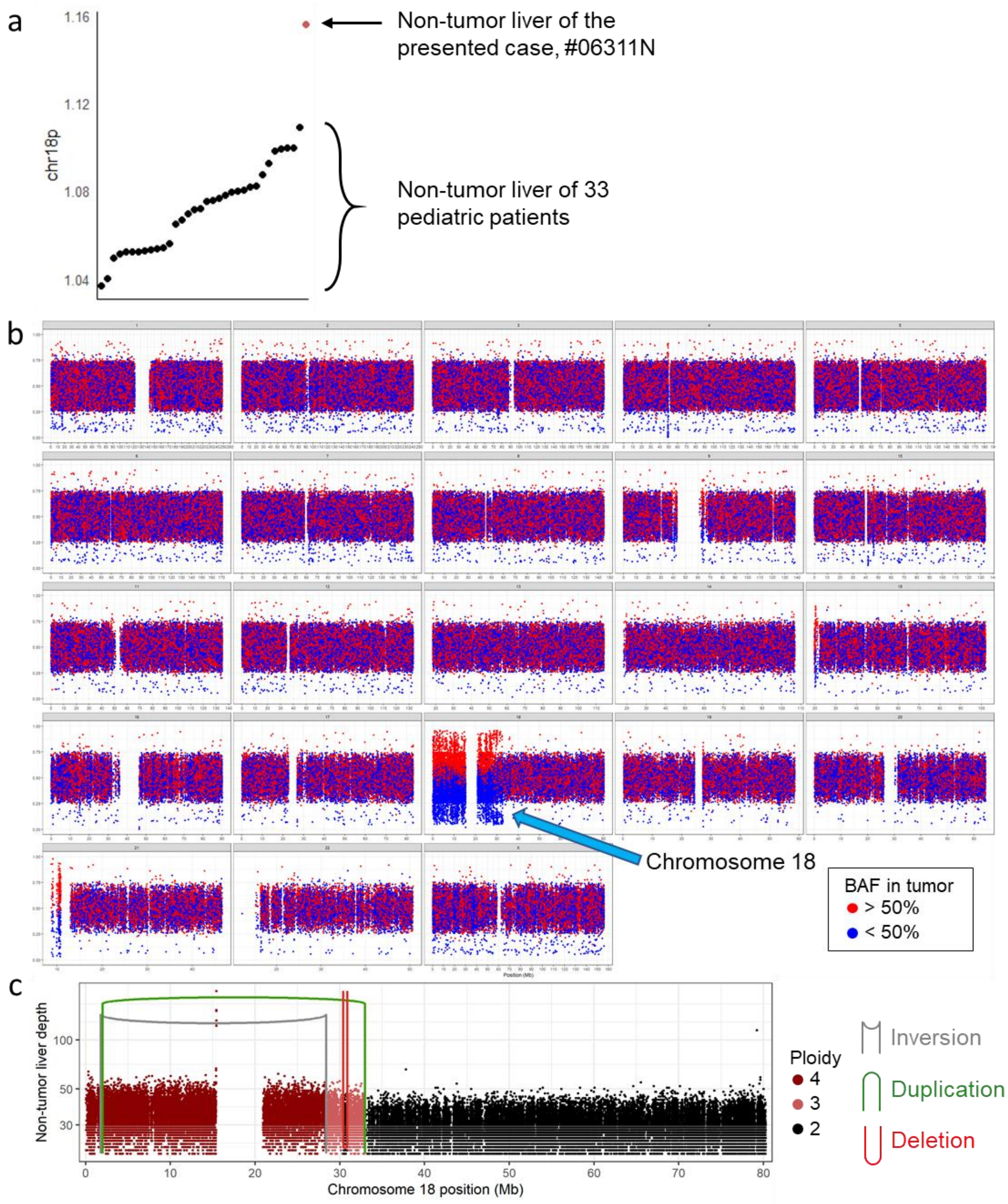

Fig. S4: Molecular proof of mosaicism in the non-tumor liver

a) Mean expression of all coding genes on the chr18p, normalized against the mean expression of all coding genes in the whole genome, in 34 pediatric non-tumor livers, including the one from the presented case. **b)** B-allele frequency (BAF) from Whole-Genome Sequencing data, showing SNP allele frequency in the non-tumor liver, split by chromosome and colored according to their BAF in the tumor (higher or lower than 50%). A disequilibrium is seen in chr18p and part of chr18q (up to 33 Mb**). c)** Sequencing depth along chromosome 18 in the non-tumor liver sample, colored according to the ploidy in the mosaic cells. The structural variants are shown in green (duplication), red (deletion) and grey (inversion).

BAF: B-allele frequency. SNP: single nucleotide polymorphism.

Supplementary Figure 5

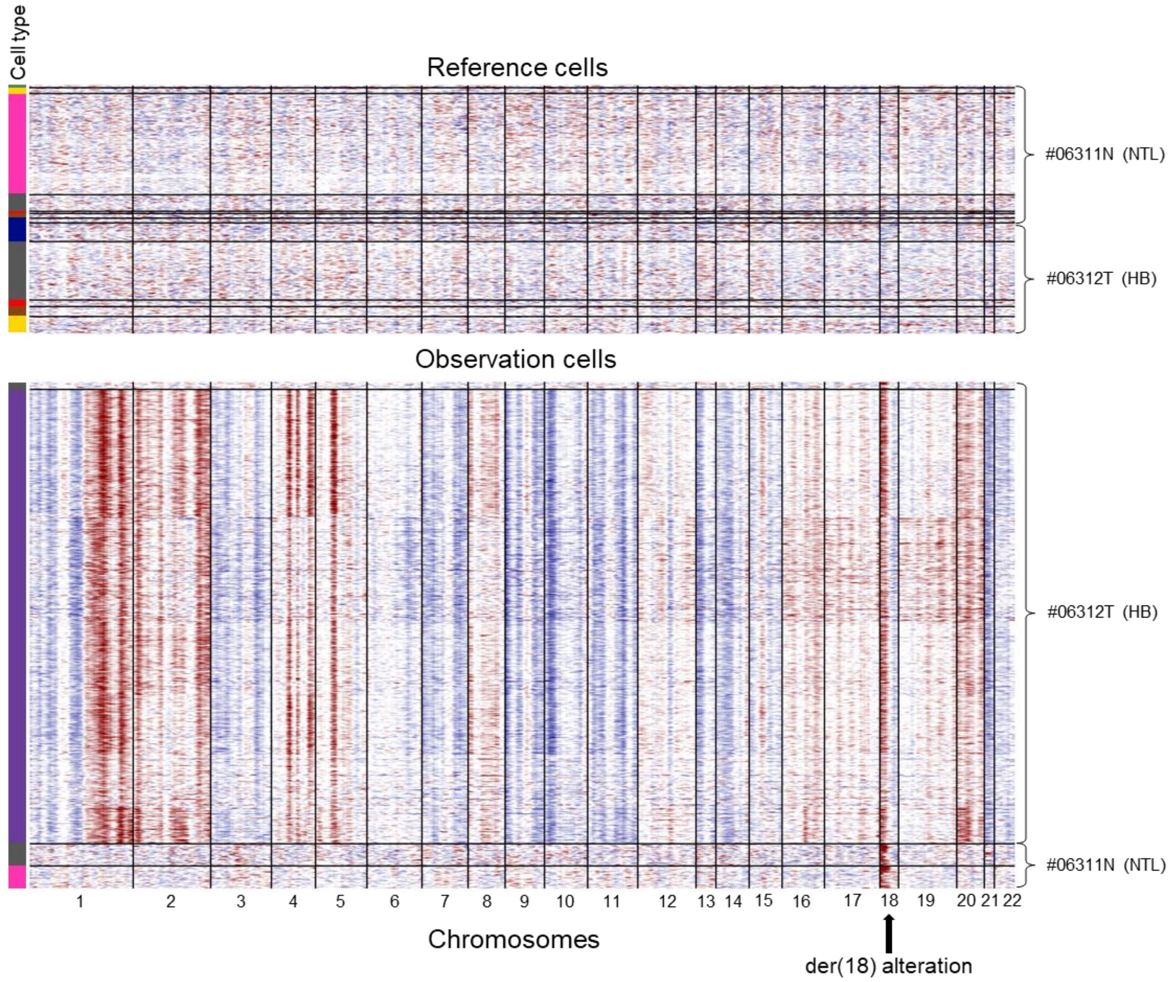

Fig. S5: Inference of copy-number from expression in single-nucleus RNAseq

Identification of copy-number alterations at the single cell level using the inferCNV tool. Each row corresponds to a cell, with the annotation of the cell type (left) and sample of origin (right). The rearranged chromosome der(18) can be seen in all tumor cells as well as in a fraction of non-tumor cells: 7% of hepatocytes, 32% of endothelial cells from the NTL sample, and 4% of endothelial cells from the HB sample.

HB: hepatoblastoma. NTL: non-tumor liver.

Supplementary Figure 6

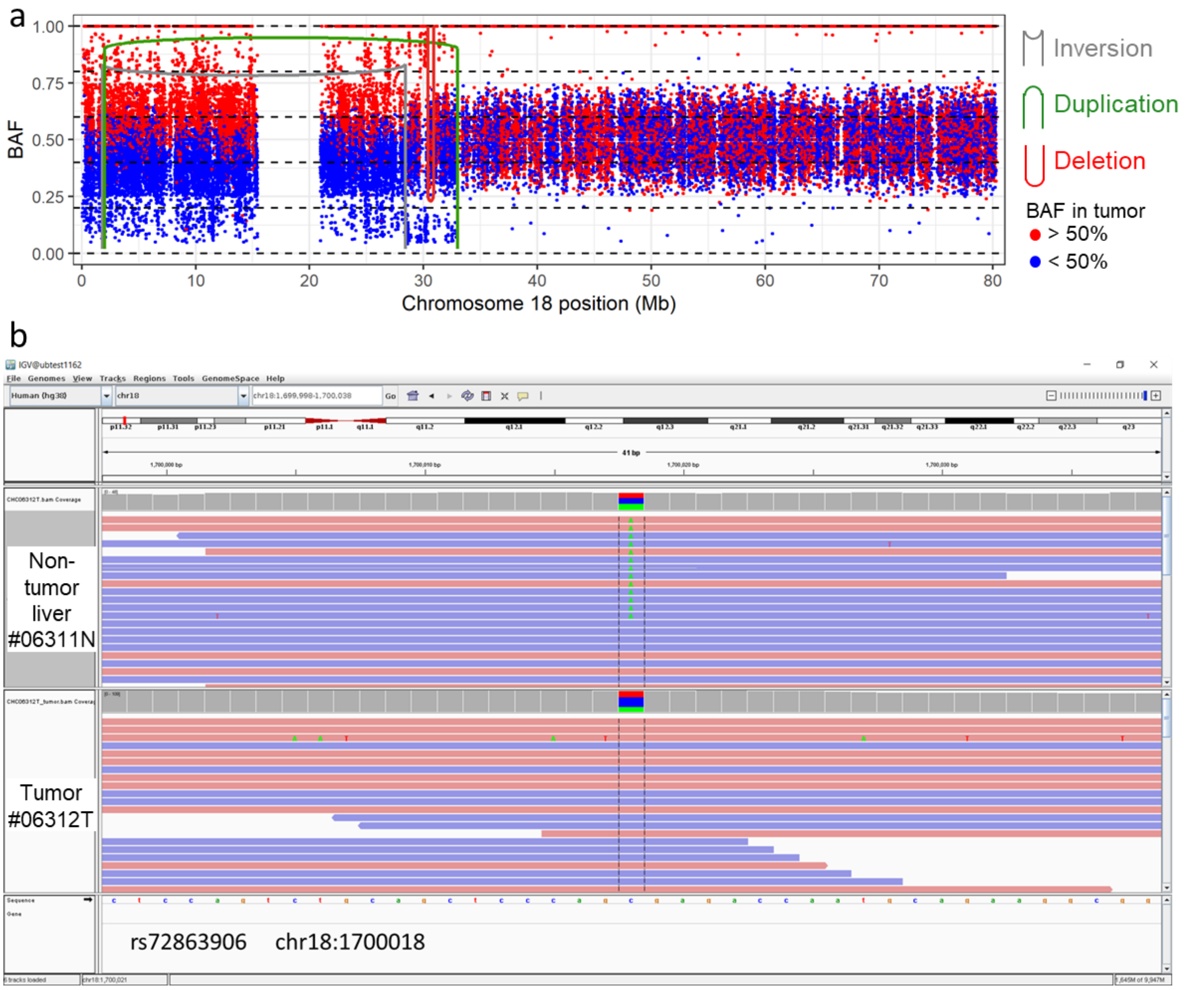

Fig. S6: Evidence of the presence of 3 haplotypes for chromosome 18

a) Unusual patterning of the B-allele frequency in the non-tumor liver (#06311N), colored according to the B-allele frequency in the tumor sample (#06312T). The structural variants are shown in green (duplication), red (deletion) and grey (inversion). **b)** One example of a multi-allelic SNP showing 3 different alleles in both the non-tumor liver and the tumor.

BAF: B-allele frequency. SNP: single nucleotide polymorphism.
