# Supplementary material for: Molecular evolution of mosaic chromosome 18 copy-number alterations from gametes to hepatoblastoma
Source: JHEP Rep. 2026 Apr 20;8(8):101862. doi: 10.1016/j.jhepr.2026.101862 (PMC13355473; doi:10.1016/j.jhepr.2026.101862)
Supplement: Multimedia component 2 [file mmc2.docx]

**JHEP Reports**

**CTAT methods**

Tables for a “Complete, Transparent, Accurate and Timely account” (CTAT) are now mandatory for all revised submissions. The aim is to enhance the reproducibility of methods.

- Only include the parts relevant to your study
- Refer to the CTAT in the main text as ‘Supplementary CTAT Table’
- Do not add subheadings
- Add as many rows as needed to include all information
- Only include one item per row

**If the CTAT form is not relevant to your study, please outline the reasons why:**

|  |
| --- |

- 1. **Antibodies**

| **Name** | **Citation** | **Supplier** | **Cat no.** | **Clone no.** |
| --- | --- | --- | --- | --- |
|  |  |  |  |  |

- 1. **Cell lines**

| **Name** | **Citation** | **Supplier** | **Cat no.** | **Passage no.** | **Authentication test method** |
| --- | --- | --- | --- | --- | --- |
|  |  |  |  |  |  |

- 1. **Organisms**

| **Name** | **Citation** | **Supplier** | **Strain** | **Sex** | **Age** | **Overall n number** |
| --- | --- | --- | --- | --- | --- | --- |
|  |  |  |  |  |  |  |

- 1. **Sequence based reagents**

| **Name** | **Sequence** | **Supplier** |
| --- | --- | --- |
|  |  |  |

- 1. **Biological samples**

| **Description** | **Source** | **Identifier** |
| --- | --- | --- |
| One patient, 4 samples from 2 different timepoints: at diagnosis (tumor biopsy + blood), at surgery (tumor + non-tumor liver) |  |  |

- 1. **Deposited data**

| **Name of repository** | **Identifier** | **Link** |
| --- | --- | --- |
| EGA (European Genome-phenome Archive) | EGAS00001008072 |  |

- 1. **Software**

| **Software name** | **Manufacturer** | **Version** |
| --- | --- | --- |
| Trailmaker | <https://app.trailmaker.parsebiosciences.com> | 1.5.0 |
| FACETS | <https://github.com/mskcc/facets> | 0.6.1 |
| R software | R Foundation for Statistical Computing <https://www.R-project.org> | 4.2.3 |
| Seurat | <https://satijalab.org/seurat/> | 4.3.0 |
| inferCNV | <https://github.com/broadinstitute/inferCNV> | 1.10.1 |

- 1. **Other (*e.g*. drugs, proteins, vectors etc.)**

|  |  |  |
| --- | --- | --- |
|  |  |  |

- 1. **Please provide the details of the corresponding methods author for the manuscript:**

| - Dr Théo Hirsch, Centre de Recherche des Cordeliers, INSERM U1138, Sorbonne Université-Inserm-Université Paris Cité. E-mail: [theo.hirsch@inserm.fr](mailto:theo.hirsch@inserm.fr) |
| --- |

**2.0 Please confirm for randomised controlled trials all versions of the clinical protocol are included in the submission. These will be published online as supplementary information.**

|  |
| --- |
